# Boiling over: A Descriptive Analysis of Drinking Water Advisories in First Nations Communities in Ontario, Canada

**DOI:** 10.3390/ijerph13050505

**Published:** 2016-05-17

**Authors:** Lindsay P. Galway

**Affiliations:** Department of Health Sciences, Lakehead University, Thunder Bay, ON P7B 5E1, Canada; lgalway@lakeheadu.ca; Tel.: +1-807-766-7280

**Keywords:** drinking water, drinking water advisories, environmental justice

## Abstract

Access to safe and reliable drinking water is commonplace for most Canadians. However, the right to safe and reliable drinking water is denied to many First Nations peoples across the country, highlighting a priority public health and environmental justice issue in Canada. This paper describes trends and characteristics of drinking water advisories, used as a proxy for reliable access to safe drinking water, among First Nations communities in the province of Ontario. Visual and statistical tools were used to summarize the advisory data in general, temporal trends, and characteristics of the drinking water systems in which advisories were issued. Overall, 402 advisories were issued during the study period. The number of advisories increased from 25 in 2004 to 75 in 2013. The average advisory duration was 294 days. Most advisories were reported in summer months and equipment malfunction was the most commonly reported reason for issuing an advisory. Nearly half of all advisories occurred in drinking water systems where additional operator training was needed. These findings underscore that the prevalence of drinking water advisories in First Nations communities is a problem that must be addressed. Concerted and multi-faceted efforts are called for to improve the provision of safe and reliable drinking water First Nations communities.

## 1. Introduction

Water is a key determinant of human health and ecosystem function and a prerequisite for sustainable development. Water also has spiritual and cultural significance for many First Nations peoples [1]. According to the World Health Organization, “(w)ater is the essence of life and human dignity” [2] (p. 3). Given the central role of water in protecting and promoting all aspects of health and wellbeing, access to safe and reliable drinking water and sanitation has been designated a human right. On 23 September 2011, a resolution was passed by the United Nations Human Rights Council (UNHRC) specifying that the right to water “entitles everyone to sufficient, safe, acceptable, physically accessible and affordable water for personal and domestic uses” [3] (p. 1). Governments were called upon to take action towards implementing this right for all its citizens. Currently, the Canadian Constitution does not explicitly recognize the right to water [4].

Access to safe and reliable drinking water is commonplace across most of Canada. However, many First Nations peoples living on reserves across the country do not have access to safe and reliable water for drinking and other household activities. Disturbing inequities exist in terms of access to water and water quality between on-reserve First Nations communities and other communities across the country [5]. For example, “the number of water-borne infections in First Nations communities is an alarming 26 times higher than the Canadian national average” [6] (p. 1) while people living on reserves are 90 times more likely to have no access to running water compared to other Canadians [5]. Following an audit of drinking water safety on First Nation reserves in 2005, the Canadian Commissioner of the Environment concluded that First Nations communities do not receive the same level of protection as the rest of the Canadian population with respect to water and drinking water [4]. In the province of Ontario specifically, *The Report of the Walkerton Inquiry* noted that the poorest-quality water in the province is found in First Nations communities [7].

The problem of inadequate access to safe and reliable drinking among First Nations populations in Canada is compounded by the underlying social, political, and economic marginalization and disadvantage faced by First Nations peoples of Canada [8]. First Nations peoples are one of, if not the most, disadvantaged population in Canada [9]. Poverty, unsafe living conditions, and poor access to health and social services has been documented in numerous reports and studies (e.g., [8,10,11,12,13,14] and manifests in inequities in health and well-being. Table 1 summarizes numerous startling statistics in terms of economic, social, and health indicators that highlight disparities (see Table 1 for examples). Recognizing the pervasive disadvantage and marginalization that First Nations peoples and communities in Canada is important for contextualizing the discussion on access to safe and reliable drinking presented in this paper.

The problem of inadequate access to safe and reliable drinking among First Nations peoples in Canada has been recognized across all levels of government and across numerous sectors resulting in policy initiatives and programs (e.g., the *First Nations Drinking Water Safety Programme* initiated in 2001 and the *First Nations Water Management Strategy* developed by Health Canada and Indigenous and Northern Affairs and implemented in 2003 [21]), legislative changes (e.g., *The Safe Drinking Water for First Nations Act* Bill S-8) [22], and funding aimed at improving water and wastewater system infrastructure (*i.e.*, $1.6 billion federal investment from 2003 to 2008 allocated through the *First Nations Water Management Strategy)*. Despite these efforts, the problem of inadequate and unreliable access to safe drinking water in First Nation communities persists [5]. The persistence of this problem is demonstrated by the large number of drinking water advisories (DWA) among First Nations drinking water systems in Canada. As of 31 December 2015 (the most recently available data at the time of publishing), there were 131 DWAs in effect in First Nations communities across Canada (excluding British Columbia, where First Nations health programming has been transferred to the First Nations Health Authority). Of these advisories 85%, or 65%, were in communities in the province of Ontario [23].

Drinking water advisories are preventative measures issued to protect the public against potential health threats from drinking water supplies and can be issued for a range of reasons including problems with drinking water equipment or microbiological contamination [24]. Health Canada utilizes three types of drinking water advisories: boil water, do not consume, and do not use advisories [25,26]. Do not consume orders are issued in cases where contaminants are present that cannot be removed through boiling while do not use orders are “issued to the public when the contaminant that poses a health risk cannot be removed from the water by boiling, and exposure to the water could cause skin, eye, and/or nose irritations or when an unknown contaminant has polluted the drinking water supply (e.g., a chemical spill)” [24]. The large majority of advisories in Canada are boil water advisories [23]. Boil water advisories “are issued when the microbiological quality of drinking water is suspected or confirmed to be compromised, meaning disease-causing micro-organisms, such as bacteria, viruses or parasites, could be in the drinking water” [26]. It is important to note that the issuance of a boil water advisory does not necessary indicate unsafe drinking water as they are often issued as precautionary measures [27]. Rather, boil water advisories reflect situations where drinking water has been deemed *potentially* unsafe. For example, boil water advisories are often issued when there is a problem with the drinking water process or distribution system such that the drinking water supply is potentially unsafe and poses a risk to public health [26]. Drinking water advisories are therefore reflective of potentially compromised drinking water and can serve as a proxy for reliable access to safe drinking water [28,29,30,31].

It is important to note that DWAs are also a common problem among small drinking water systems serving non First Nations communities, particularly those located in rural and remote locations (a small system is defined as a drinking water systems serving less than 5000 people according to Health Canada) [26]. Broadly speaking, small drinking water systems face a number of unique challenge compared to larger drinking water systems: they often use untreated surface water, lack access to funding, face personnel and capacity issues, and use aging distributions systems [26,30].

Although there are limitations in using DWA data as a proxy for reliable access to safe drinking water (e.g., inconsistent reporting behavior, the precautionary nature of advisories, reporting timeliness), very few measures are available to monitor progress towards improving access to safe and reliable drinking water in First Nations communities [32]. This paper describes trends and characteristics of DWAs in First Nations community drinking water systems across Ontario over a 10-year study period.

## 2. Materials and Methods

### 2.1. Study Design

A cross-sectional study design was used to describe trends and characteristics of DWAs in First Nations drinking water systems in Ontario issued between 1 January 2004 and 31 December 2013.

### 2.2. Study Area

Ontario is Canada’s most populous province with an estimated population of 13.6 million [33]. According to the 2006 census, 242,495 of the more than 1.1 million Canadians who identified themselves as Aboriginal live in the province of Ontario [34]. There are 126 First Nations communities recognized by the federal government in the province, many are located in rural and remote locations in Northwestern Ontario [34]. Of the 127 First Nation communities in Ontario, 113 had an on-reserve population of less than 1000 [35]. In 2009–2010, the time of a province wide assessment of First Nations drinking water systems in the province, there were a total of 158 water systems [36].

It is important to note that there is substantial heterogeneity across First Nations communities within the province.

### 2.3. Data Sources and Variables

The data used for this study consisted of all DWAs issued for First Nations drinking water systems across Ontario and reported by Health Canada from 2004 through to 2014. 2004 was selected as the beginning of the study period because reporting is inconsistent in earlier years [24]. Attributes of each advisory in the dataset included: district where the DWA was issued, name of the First Nation community where the DWA was issued, name of the drinking water system under advisory, type of drinking water advisory, type of drinking water system under advisory (*i.e.*, community or private system), reason(s) for issuing the advisory, the start date of the advisory, and the end date of the advisory. Because this paper focused on DWAs in community drinking waters systems, those advisories issued in private drinking waters systems were excluded as a result. A total of 39 DWAs (3.9%) were excluded from the dataset. The reasons for issuing a DWA as reported by Health Canada included: (1) “significant deterioration in source water quality”; (2) “equipment malfunction during treatment or distribution”; (3) “inadequate disinfection or disinfectant residuals”; (4) “unacceptable microbiological quality”; (5) “unacceptable turbidities or particle counts”; (6) “operation of system would compromise public health”; and (7) “unknown”. More than one reason could be coded for a single DWA; 19% of the DWAs in the dataset were assigned more than one reason.

The advisory start date, defined as the date the DWA was issued, was used to derive the month, year, and the season of the advisory. Subsequently, monthly and yearly time series representing the number of cases per month and the number of cases per year over the 10-year study period was generated. A categorical variable for “season” was also created indicating whether the DWA occurred in winter (December, January, February), spring (March, April, May), summer (June, July, August) or fall (September, October, November). It should be noted that using the start date of the advisory to count the number of advisories ensures that there are no carry over effects in the annual counts from year to year. A continuous variable indicating the duration of each DWA in terms of the total number of days the event was in effect during the study period was generated by calculating the difference between the start date and the end date of each advisory. For those DWA that were ongoing at the time of analysis, 31 December 2013 was used as the end date to calculate the duration of the DWA during the study period. The continuous variable indicating duration of the DWA was then used to generate a categorical variable measured on three levels: DWA in effect less than 1 month, DWA in effect between 1 month and 1 year, and DWA in effect more than year.

Characteristics of individual drinking water systems were linked to each DWA in the dataset using data reported in the 2011 *National Assessment of First Nations Water and Wastewater Systems—Ontario Regional Roll-Up Report.* This report summarizes results from a national, independent assessment implemented as part of the *First Nations Water and Wastewater Action Plan* (FNWWAP) implemented in 2008. The FNWWAP funded the construction and renovation of water and wastewater facilities and operator training and also implemented *The National Assessment of First Nations Water and Wastewater Systems.* The purpose of the national assessment was to define “deficiencies and operational needs of water and wastewater systems, to identify long-term water and wastewater needs for each community and to review sustainable, long-term infrastructure development strategies” [37]. The Ontario regional report provides a large amount of individual drinking water system characteristics for all of the systems that were assessed as part of the assessment process. Between 2009 and 2011, 99% of First Nations communities in Ontario participated in the assessment. A selection of system characteristics were extracted from the report and linked to individual DWA events for the purpose of this study. Specifically: water source (groundwater, surface water, groundwater under the influence of surface water, or unknown), drinking water system construction year, population served by the drinking water system, treatment system classification level, and treatment system training held by the primary operator. The data extracted from the report were “cleaned” (*i.e.*, assessed for data entry errors and missingness) and linked to individual DWAs by drinking water system name. This linking was done manually to ensure quality and completeness and because spelling of water system names sometimes differed across the Health Canada DWA data and the *National Assessment of First Nations Water and Wastewater Systems* report. Finally, a binary variable indicating whether the primary operator was sufficiently trained for the drinking water treatment system was generated by comparing the treatment classification level of the system (the classification level reflects the complexity of the treatment systems) to the level of training held by the primary operator of the system.

### 2.4. Data Analysis

A suite of visual and statistical tools were used to describe the DWA data in general, to describe the temporal trends, and to summarize the characteristics of the drinking water systems in which the DWAs were issued.

Characteristics of the DWAs in general and the reasons for issuing DWAs were summarized using tables and counts. An annual plot was generated to illustrate the changes in the number of DWAs across the study period. Note that the annual graph does not include carry over effects from year to year because issue date was used to count the yearly totals. Total counts, percentages of total counts, and means were used to describe the monthly and seasonal trends. Poisson regression models were used to test the statistical significance of monthly, yearly, and seasonal peaks in the DWA time series data. Since monthly, yearly, and seasonal DWA events are indicated by count data, a Poisson regression model was selected. The Poisson distribution is commonly used to model count and rate data [38]. For the purpose of this study, a *p*-value of less than 0.05 was considered statistically significant. The year, month, and season with the lowest number of cases were used as the reference category [39]. Characteristics of the drinking water systems in which the DWAs were issued were summarized using tables and descriptive statistics.

Analyses were conducted in the R statistical computing environment (version 3.2.3) (R Foundation for Statistical Computing, Vienna, Austria) [40].

## 3. Results

### 3.1. General

A total of 402 DWAs were issued in First Nations community drinking water systems in Ontario between 1 January 2004 and 31 December 2013. Of these, 13 (3%) were do not consume orders and 389 (97%) were boil water advisories. There were no reported do not use orders during the study period. It is also noteworthy that an additional 13 DWAs were in effect during the study period, but were issued prior to 2004, and that 48 DWAs were in effect at the end of the study period.

**Figure 1:**
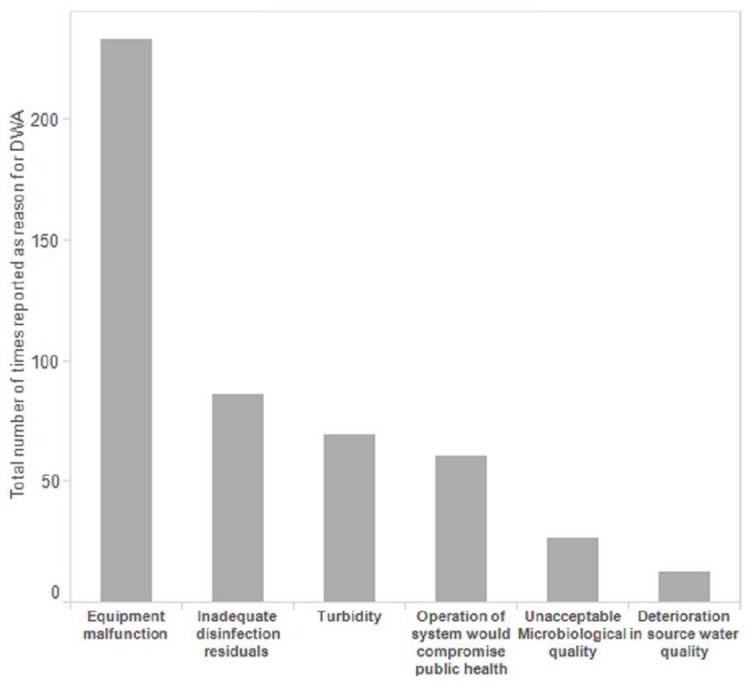
Reasons for DWAs in First Nations drinking water systems in Ontario, 2004–2013.

Figure 1 summarizes the reasons for issuing DWAs reported in the dataset. “Equipment malfunction” was the most commonly reported reason (*n* = 233). “Inadequate disinfection residuals” (*n* = 86) and “Unacceptable turbidities or particle counts” (*n* = 69) were the second and third most commonly reported reasons. Nearly all do not consume advisories (11 of 13) were issued because “Operation of the system would compromise public health”.

The average advisory duration across all advisories in the province during the 10-year study period was 294 days. The total number of DWA days during the study period was 118,307. Nearly 20% of all DWA events were medium term advisories in effect between 1 month and 1 year; 14% were long-term advisories in effect for more than 1 year.

### 3.2. Temporal Trends in DWAs

**Figure 2:**
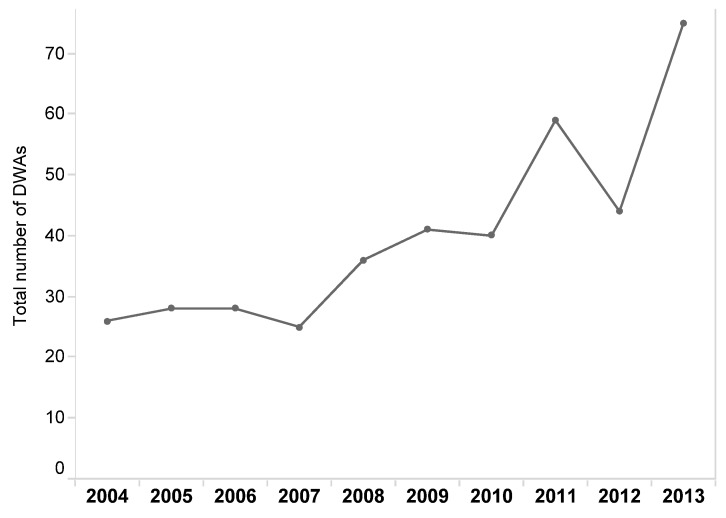
Annual trends in DWAs in First Nations drinking water systems in Ontario, 2004–2013.

The number of DWAs increased across the study period. The total number of DWAs issued across all years in the study period is shown in Figure 2. The number of reported DWAs per year varied from 25 to 75. The years with the greatest number of DWAs were 2013 (19%) and 2011 (15%). More than half of all DWAs (52%) were issued between 2011 and 2013. The peaks identified in 2011 and 2013 are both statistically significant (*p* < 0.05). DWAs were issued in all months and all seasons, but most occurred in July (14%) and in the summer months (32%) reflecting seasonality in the occurrence of DWAs. The peak in the number of DWAs in July and in the summer months are both statistically significant (*p* < 0.05). Total counts and the percentage of total counts for all months and seasons are reported in Table 2.

### 3.3. Characteristics of Drinking Water Systems with DWAs

One DWA was reported in 52 drinking water systems while more than one advisory was reported in 58 drinking water systems; therefore 70% of all First Nations community drinking water systems were affected by at least one DWA during the 10-year study period. The large majority of advisories were issued in drinking water systems supplied by surface water (78%). Most advisories (85%) occurred in drinking water systems that were constructed more than 10 years ago and in systems serving less than 500 people. Across all 402 DWAs reported, almost half (47%) occurred in drinking water systems where the primary operator did not have adequate training for the treatment system (See Table 3).

## 4. Discussion

The provision of safe and reliable drinking water among First Nations communities is a priority public health and environmental justice issue across Canada. Using DWAs as a proxy for reliable access to safe drinking water, this paper provides further evidence for the magnitude and urgency of this problem. The DWA data indicate that more than 400 advisories were issued in First Nations community drinking water systems between 2004 and 2013, leading to a total of 118,307 advisory days. A small percentage (3%) of the DWAs were do not consume orders which are issued in cases where contaminants that cannot be removed through boiling are present [24]. It is particularly troubling that 14% of the DWAs were long term advisories, in effect for more than one year with some in effect for the entire 10-year study period. This study shows that access to safe drinking water is not reliable within First Nations communities across Ontario; it is sporadic in most cases and chronically unavailable in far too many.

When a boil water advisory is issued, community members are advised to boil water for one minute prior to use including drinking water, for ice, brushing teeth, food preparation, infant formulas *etc.* [41]. In cases where water cannot be boiled, community members are directed to disinfect the water using household bleach, or to purchase commercially packaged water [41]. Boiling water prior to use quickly becomes a significant burden in terms of time, particularly during long term advisories and for vulnerable sub-populations within First Nations communities, such as the chronically ill, pregnant mothers, children, and the elderly [42]. Purchasing bottled water for months and years is clearly an important economic burden. A report by the Polaris institute in 2008 investigated and documented the experience of living in a First Nation community under long-term boil water advisory in Canada [43]. The Polaris institute report draws attention to the lived experiences and wide ranging impacts that emerge in communities facing long term DWAs such as: the economic injustice of having to purchase expensive bottled water, resorting to drinking untreated lake water when bottled water cannot be accessed, increased risk of illness and disease, the burden and loss of time resulting from boiling water before consumption, and the constant underlying anxiety about poor drinking water quality. The prevalence of DWAs in First Nations communities in Canada is “a national disgrace” [43] that clearly results in myriad consequences that are particularly problematic for an already marginalized and disadvantaged group.

The prevalence of DWAs has increased since 2004; 2013 was the year with the greatest number of DWAs in First Nations drinking water systems. This increasing trend may, in part, reflect increased monitoring and surveillance and/or increased drinking water sampling over this same time period. Since water quality and safety issues are more likely to be detected, and DWAs issued when water quality monitoring is enhanced [24,44]. It is unlikely, however, that increased monitoring, surveillance, or water sampling would fully explain the increasing trend in DWAs reported in this paper [24]. Further, Health Canada has documented that the reporting of DWA has been consistent since 2003 such that changes in reporting behavior are unlikely to have had an effect on the trends reported here [24]. These findings provide evidence that limited progress has been made towards the provision of safe and reliable drinking water for First Nations communities despite monetary and infrastructural investments by the federal government on the order of $2 billion over the last decade [28].

It is also noteworthy that a large number of DWAs were issued due to high levels of turbidity, *i.e.*, a measure of the cloudiness of water caused by particles of clay, silt, organic and inorganic matter, and microscopic organisms [26]. High levels of turbidity has been shown to reduce the effectiveness of water treatment and may also provide a medium for microbial growth in source waters and increase the risk of waterborne illness [45,46,47]. Turbidity is often caused by spring runoff and extreme rainfall events and may contribute to the seasonality seen in the DWA data [47,48]. Seasonality, the summertime peak in the occurrence of DWAs specifically, may also be caused by increased summer time temperatures and lower water levels in source water, which have been documented as risk factors for poor water quality and waterborne disease outbreaks in other research in Canada [24,42,49]. The majority of advisories were reported in surface water, which are more likely to be influenced by turbidity and the effects of temperature, spring runoff, and heavy rainfall events [50]. Future research should examine the relationship between weather and hydroclimatic variability, turbidity, and DWA events specifically. Taken together, the important role of turbidity as a causative factor for issuing DWA and the evidence for seasonality in the DWA likely indicate the “upstream” effects of source water quality deterioration, particularly amoung those communities using surface water sources.

Equipment malfunction was the most commonly reported reason for issuing a DWA advisory in First Nations community drinking water systems. Health Canada has reported that problems with drinking water equipment are also the most commonly reported reason for all boil water advisories reported in Canada (data which includes the DWAs occurring in First Nations water systems and reported here) [26]. The large number of DWAs issued due to equipment malfunction highlights that enhanced funding directed towards drinking water (and wastewater) system construction, renovations, and upgrade programs are needed. The *Canadian Center for Policy Alternatives* has argued that an annual investment of $470 million over the next decade is needed to improve on-reserve drinking water provision across Canada [51]. Given that a large proportion of DWAs occurred in systems where the primary operators required more training, operator training and capacity building are also high priorities for funding. Water system operators are members of the First Nation communities served by the water system such that operator training and capacity building programs should draw on the emerging literature around cultural safety (e.g., [52]) while also acknowledging the lasting effects of colonization given that water-related issues in First Nations communities are related to “broader historical and unjust relationships between Aboriginal peoples and the Crown” [53] (p. 4).

However, reducing the prevalence of DWAs, and making sustainable progress towards the provision of reliable and safe drinking water in First Nations communities, requires more than additional funding and more than technical fixes at the water system level [53]. Over the last decade, government action aimed at addressing water-related issues in First Nations communities has been characterized by a narrow approach aimed almost exclusively at technological and infrastructural interventions [28]. This narrow approach will no longer suffice. We must acknowledge that the characteristics and trends in DWAs are reflective of a complex web of interconnected social, cultural, economic, political, and ecological factors and that the lack of access to safe and reliable drinking is a “multi-layered problem” [53] (p. 4) [54]. Moreover, water-related issues are just one of many pressing issues facing First Nations communities in Ontario and beyond. We must therefore widen our view, and expand our efforts, from a focus on technical fixes towards a broader approach addressing issues related to water governance and acknowledging the social and ecological determinants of water provision, safety, and quality [55,56,57]. Researchers studying other marginalized and disadvantaged communities within developed nations have come to similar conclusions regarding the need to move beyond technical fixes and account for the role of social and ecological factors within the realm of water provision, safety, and quality (e.g., [58,59]). With regards to First Nations in Canada, fragmentation and source water protection are two priority water governance issues that should be addressed.

The fragmented nature of drinking water provision and water management is a growing concern in Canada and has been highlighted as a primary underlying determinant of water-related issues in First Nations communities [4,54,60]. It should be noted that provincial regulatory water standards do not apply to on-reserve First Nations communities [61] and that “(t)he governance and management structures of drinking water in First Nations and non-First Nations are different” [56] (p. 1). Currently, the management of water and the provision of drinking water is divided between Indigenous and Northern Affairs Canada (INAC), Health Canada, and Environment and Climate Change Canada (Environment Canada prior to 2016) and First Nations communities themselves [57]. Specifically, INAC “…provides advice and funding assistance for the design, construction, operation, and maintenance of water and wastewater system [62] (p. 5). Health Canada works with First Nations communities to identify overall drinking water quality and provides guidance about quality and safety issues (including advice regarding DWAs). Environment and Climate Change Canada provides advice and guidance material in the areas of source water protection and sustainable and also provides First Nations communities with advice and guidance regarding source protection and water use [57]. In 2006, an Expert Panel on Safe Drinking Water for First Nations reported that “(t)hese arrangements are neither comprehensive nor easily deciphered; most critically, there are numerous gaps and a lack of uniform standards, as well as enforcement and accountability mechanisms” [63] (p. 1). First Nations communities themselves are responsible for the delivery of water to community members and the design, maintenance, and operation of the water systems [61]. There is a pressing need to more fully understand the barriers and challenges of this patchwork approach and to improve communication and collaboration amoung the multiple agencies that share responsibilities regarding the provision and management of drinking water for First Nations communities.

Improved water governance also requires more explicit attention to source water protection at the watershed scale [31]. As Phare has argued, and I would agree, “one solution to the drinking water crisis is to “go upstream”, that is, to protect the drinking water source” [54]. The importance of source water protection has not been adequately accounted for in regulatory efforts and policy and action to date [64]. As an example, the *Safe Drinking Water for First Nations Act* generally overlooks the fact that on-reserve drinking water quality and quantity is heavily impacted by off-reserve activities that affect source waters such as resource extraction, forestry practices, and agriculture [22,55]. Another, rather shocking example of lacking attention to upstream processes and factors, the water treatment plant in one reserve community in Northern Ontario was built downstream from its sewage lagoon [64]. Source water protection plans at the watershed level, with meaningful engagement with on-reserve populations, that are informed by First Nations‘ traditional views on water and indigenous ways of knowing and living, and that respect treaty rights are sorely needed [5,60,65]. Patrick has argued that source water protection can offer co-benefits such re-connecting health and place within First Nations communities [28].

Finally, we must also accept that there is no “one-size-fits all” solution to the problem of DWAs or the lack of reliable access to safe drinking water among First Nations communities. Rather, we must recognize, accommodate, and respect the heterogeneity that exists across First Nations communities, both within Ontario and across the country [66]. Efforts aimed at addressing this problem should build on the unique strengths of individual First Nations communities while addressing local vulnerabilities to co-develop sustainable and effective action plans.

This study has limitations that should be acknowledged when interpreting the results. An important limitation is that all information about the drinking water systems was obtained from the *National Assessment of First Nations Water and Wastewater Systems - Ontario Regional Roll-Up Report* [25]. The drinking water system information in this report was collected by Neegan and Burnside for as part of the FNWWAP. It is impossible to discern whether there are any errors in data collection and reporting. Further, these data were collected during site visits that took place in September and October of 2009 and in May through September of 2010. It is therefore possible therefore that changes and upgrade were made to individual drinking water systems since the data was collected that are not captured in the data reported in this paper [36]. It is likely that the number of advisories reported in this study is an underrepresentation of situations in which DWA are warranted since Health Canada advises First Nations to issue DWA but issuing and reporting a DWA is not mandatory [67,68]. Although Health Canada has stated that the consistency of reporting of DWA events has improved significantly since the implementation of the *First Nations Water Management Strategy* in 2003, it is still possible that there are inconsistencies in terms criteria used for issuing an advisory or the reporting of DWA events. It is however, unlikely that any inconsistencies would significantly influence the study results. An additional noteworthy limitation is that remoteness of communities has not been directly examined. Although remoteness of the drinking water systems under advisory would have been a relevant system characteristic to consider, this data was not included in the present analysis. Finally, it is important to acknowledge that it is possible that the increasing trend in DWA events during the study period reported here could reflect enhanced monitoring and surveillance efforts during this same time period.

Despite these limitations, this study has generated new knowledge and makes relevant contributions to our currently limited understanding of an important issue in Canada—access to safe and reliable drinking water for First Nations communities. This paper also makes progress towards addressing the current shortfall in available of “progress indicators” that can be used monitor improvements in terms of water quality, safety, and provision among Canada’s First Nations populations that has been highlighted by Morrison *et al.* [5]. However, more research and continued monitoring and surveillance of the trends and characteristics of DWAs in Canada’s First Nations communities is needed to ensure that policy, monetary investments, and regulatory changes result in lasting positive effects in terms of access to safe and reliable drinking water.

## 5. Conclusions

Inadequate access to safe and reliable drinking water among First Nations peoples is a priority public health and environmental justice issue. This paper describes trends and characteristics of drinking water advisories, used as a proxy for reliable access to safe drinking water, among First Nations communities in the province of Ontario. Although the occurrence of DWAs in First Nations communities has garnered significant media and political attention recently, this is the first paper in the peer-reviewed literature systematically describe and summarize DWA data. Overall, 402 drinking water advisories were issued during the 10-year study period; nearly all were boil water advisories. The annual number of advisories increased significantly over the study period. The average advisory duration was 294 days, resulting in a total of 118,304 advisory days across the study period. Most advisories were reported in summer months and equipment malfunction was the most commonly reported reason for issuing an advisory. Many advisories were long term (in effect for more than one year). Nearly half of all advisories occurred in drinking water systems where additional operator training was needed underscoring the continued need for training and capacity building programs.

Canada’s recently elected Prime Minster, Justin Trudeau, has called the prevalence of boil water advisories among drinking water systems on First Nations reserves “a top priority” and a problem that “has gone on for far too long” [69]. Trudeau has also vowed to “end boil-water advisories on First Nations reserves” within five years of forming his government [69]. In March of 2016, the Trudeau government released a federal budget outlining $8.4 billion worth of spending commitments to Indigenous communities. Of this $8.4 billion, $1.8 billion over five years will be directed at water system and waste water system infrastructure [70]. Nevertheless, the problem of inadequate access to safe and reliable drinking water among First Nations communities in Canada will likely persist, without enhanced monetary and infrastructural investments and multi-faceted and integrated efforts towards improving water governance and source water protection at the watershed level. This paper serves as a baseline for tracking progress on Trudeau’s promise to end boil water advisories on First Nations reserves in the context of Ontario as well as progress towards the right to safe and reliable drinking water for the First Nations peoples of Canada.

## Figures and Tables

**Table 1:** Snapshot of disparities: Social, economic, and health indicators among First Nations peoples and non-First Nations people in Canada.

Indicator	First Nations People (Year) [Reference]	All Canadians (Year) [Reference]
Age standardized prevelance of type 2 diabetes amoung adults	19.7% (2006) [14]	4.9% (2006) [14]
Unemployment rate	25% (2006) [15] *	6.6% (2006) [15]
Young adults (20–24) that have not completed high school	61% (2006) [16] *	13% (2006) [16]
Adults living in overcrowded housing conditions	23.4% (2008) [17]	7% (2011) [18]
Adults reporting that home is in need of major repair	37.3% (2008) [17]	9.1% (2010) [19]
Average household income ^$^	$18,962 (2006) [20] *	$27,097 (2006) [20]
Life expectancy, Males	68.9 years (2001) [8]	76.3 years (2001) [8]
Life expectancy, Females	76.6 years (2001) [8]	81.1 years (2001) [8]

* Living on reserve only; ^$^ Aboriginal, which includes metis and Inuit in addition to First Nation peoples [20].

**Table 2:** Monthly and seasonal counts of DWAs issued in First Nations community drinking water systems in Ontario, 2004–2013.

Variable	Total Count	Mean (Min–Max)	%
*Month*			
January	19	2 (0,5)	4.73
February	32	3 (0,6)	7.96
March	25	2 (0,4)	6.22
April	38	4 (1,9)	9.45
May	27	3 (0,9)	6.72
June	41	4 (1,10)	10.20
July	52 *	5 (1,11)	12.94
August	36	4 (0,8)	8.96
September	36	4 (2,9)	8.96
October	31	3 (0,7)	7.71
November	40	4 (1,11)	9.95
December	25	2 (0,5)	6.22
*Season*			
Winter	76	7 (1,13)	18.91
Spring	90	9 (3,21)	22.39
Summer	129 *	13 (8,27)	32.09
Fall	107 *	10 (5,24)	26.62

* = statistical significance (*p*-value < 0.05) compared to the reference month (January) and season (winter). NB: The seasons are defined as winter (December, January, February), spring (March, April, May), summer (June, July, August) or fall (September, October, November).

**Table 3:** Percentage of DWAs by water system characteristics in First Nations community drinking water systems in Ontario, 2004–2013.

Variable	Total Count	%
*Population served*		
Unknown	13	3.23
less than 500	238	59.20
500 to 1000	99	24.63
1000 to 5000	52	12.94
*Primary operator training*		
Unknown	23	5.70
Primary operator did not have adequate training for the treatment system	190	47.30
Primary operator did have adequate training for the treatment system	47	47.00
*Age of drinking water system*		
Unknown	13	3.23
1 to 10 years	47	11.69
11 to 20 years	217	53.98
21 to 30 years	117	29.10
more than 30 years	8	2.00
*Water Source*		
Unknown	11	2.73
Groundwater under the influence of surface water	26	6.47
Groundwater	52	12.94
Surface Water	313	77.86

## Abbreviations

The following abbreviations are used in this manuscript:

DWA	Drinking water advisory
FNWWAP	First Nations Water and Wastewater Action plan
AANDC	Aboriginal Affairs and Northern Development Canada
